# Tailored Cigarette Warning Messages: How Individualized Loss Aversion and Delay Discounting Rates Can Influence Perceived Message Effectiveness

**DOI:** 10.3390/ijerph181910492

**Published:** 2021-10-06

**Authors:** Hollie L. Tripp, Justin C. Strickland, Melissa Mercincavage, Janet Audrain-McGovern, Eric C. Donny, Andrew A. Strasser

**Affiliations:** 1 Department of Psychiatry, Perelman School of Medicine, University of Pennsylvania, Philadelphia, PA 19104, USA; melmer@pennmedicine.upenn.edu (M.M.); audrain@pennmedicine.upenn.edu (J.A.-M.); strasse3@pennmedicine.upenn.edu (A.A.S.); 2 Department of Psychiatry and Behavioral Sciences, School of Medicine, Johns Hopkins University, Baltimore, MD 21224, USA; jstric14@jhmi.edu; 3 Department of Physiology and Pharmacology, Wake Forest School of Medicine, Winston-Salem, NC 27157, USA; edonny@wakehealth.edu

**Keywords:** loss aversion, framing, cigarette warning messages, delay discounting, perceived message effectiveness

## Abstract

Current text-only cigarette warning labels (long-term, loss-framed messages) may not motivate positive changes in smoking behavior. The current project was a cross-sectional study examining the effects of tailored cigarette warnings on perceived message effectiveness (PME) in adult smokers (*n* = 512) conducted using Amazon Mechanical Turk (M-Turk) in January–February 2020. Participants were an average age of 40.7 (SD = 11.6), with the majority of the sample being female (62.2%) and White (88.9%). Participants reported smoking an average of 14.6 cigarettes/day (SD = 9.2) with an average FTND score of 4.6 (SD = 2.2). Participants were asked to complete a tobacco use history questionnaire, and mixed gambles and delay discounting tasks before random assignment to one of five message groups. The groups were based on a 2 (gain versus loss framing) ×2 (short-term versus long-term framing) between-subject design; a fifth group served as the control group. All experimental messages reported higher PME scores than the control (*p* values < 0.001, Cohen’s *d* = 1.88–2.48). Participants with shallower delayed reward discounting and lower loss aversion rates reported higher total PME scores, *p* values < 0.05. Our findings also suggest that loss aversion rates vary widely among smokers and that individuals are more responsive to messages congruent with their behavioral economic profile. Specifically, smokers who viewed messages congruent with their loss aversion and delay discounting rates reported higher PME scores than those who viewed incongruent messages (*p* = 0.04, Cohen’s *d* = 0.24). These preliminary findings suggest that anti-smoking campaigns may best impact smokers by tailoring messages based on individual loss aversion and delay discounting rates versus a one-size-fits-all approach.

## 1. Introduction

In 1965, Congress passed the Federal Cigarette Labeling and Advertising Act, requiring that cigarette packaging have text warning labels alerting consumers of the potential negative health consequences of smoking. In 1981, additional warnings with more descriptive text were added to the initial caution message. Since then, researchers have examined how the manipulation of text warning label size, color, placement, and congruency with images influences smokers’ decision making [1,2,3,4].

Prospect theory has been used to conceptualize how cigarette-related health messages influence risky decision making. Prospect theory presents two primary changes to previous decision-making theories directly relevant to risk messaging: framing and loss aversion [5]. Specifically, when presented with a decision, individuals’ choices are influenced by whether the outcome was conveyed as a potential gain or loss (i.e., framing), and whether people are more sensitive to the prospect of a loss than a gain (i.e., loss aversion) [6,7,8,9]. Many studies have examined the influence of gain and loss messaging on the behaviors of cigarette smokers. Some studies have found greater intentions to quit or changes in attitudes towards smoking cessation for participants exposed to gain-framed messaging [10,11,12,13]. These studies suggest that gain-framed messages are better received when individuals are trying to prevent the occurrence of disease, as is the case with smoking. However, other studies have found loss-framed messages to be more effective than gain-framed messages at increasing intentions to quit among smokers, in smokers with high self-efficacy, or for smokers simultaneously exposed to graphic warning labels [14,15,16]. One study found that utilizing *both* types of frames within a contingency management program was effective but led to different outcomes; individuals exposed to loss-framed incentives were more likely to initiate cessation and gain-framed incentives were more likely to maintain cessation [17]. However, none of these studies were conducted with consideration that loss aversion rates—measures of sensitivity to prospective losses compared to prospective gains—may vary. Based on the pattern of results in the existing literature, it is plausible that individual variation in loss sensitivity may help to understand the interaction of message type and individual or smoking group. If an individual is not loss averse, they may find loss-framed messages less compelling than someone who is loss averse.

The temporal context is another feature of framing that can influence perceptions of anti-smoking messages and warnings [18,19]. Delay discounting describes the devaluation of an outcome by the delay to its occurrence, often resulting in a preference for a short-term outcome over a long-term (but more desirable) outcome [20,21,22]. Cigarette warning labels often present distal health consequences of tobacco use, mitigating the urgency to quit smoking because of the devaluation of those future consequences [23,24,25]. It follows that those who discount the future more steeply may respond to messages with short-term framing more readily than messages with long-term framing. For example, an individual with a high delay discount rate may not be as concerned by a message indicating that they may develop lung cancer later in life as someone who discounts future health outcomes less.

Some studies have simultaneously examined the delay (short-term versus long-term) *and* sign (gain versus loss-framing) of cigarette warning labels [16,26]. One study found that gain messages improved intention-to-quit rates over loss messages, particularly for messages that expressed short-term outcomes [26]. However, another study found loss messages to have a greater impact on perceived effectiveness than gain messages, and no difference between short- and long-term messages [16]. The reason for mixed findings regarding the optimal message framing and temporal context to influence smokers’ perceptions may be because these studies did not consider how individual differences in loss aversion and/or delay discounting may influence a person’s perceptions of and reaction to these messages. This may be especially true of people who use drugs, for whom the evidence suggests lower levels of loss aversion and higher delay discounting rates than healthy populations [27,28,29,30]. Therefore, long-term, loss-focused cigarette warning labels may be less effective for influencing smokers than posited, and substantial individual variability may exist in the effectiveness of messages.

This randomized experimental study aims to evaluate the interaction between the framing of messages and person-specific behavioral economic characteristics. Specifically, the study frames anti-smoking messages by temporal context (long-term or short-term) and sign (gain or loss) and then examines whether individual loss aversion and delay discounting rates influence perceived message effectiveness.

The findings may optimize the communication of public health warnings to targeted audiences.

## 2. Materials and Methods

### 2.1. Sample

Participants were recruited from the crowdsourcing website Amazon Mechanical Turk (MTurk). A brief description of the survey was posted to the MTurk forum. The description was visible only to those who resided in the United States, had an approval rating of at least 99%, had completed at least 100 MTurk tasks, were at least 18 years of age, and had not already completed this survey. The task description did not indicate that this survey was about tobacco use, to minimize potential bias in responses to questions related to inclusion criteria. Interested individuals clicked on the Qaultrics link for the pre-screening survey, where CAPTCHA was used to prevent data entry by bots. Participants then accepted consent language before completing the one-minute screener survey to determine if they were eligible by being a current, daily smoker (i.e., had smoked 28 out of the past 30 days). Those who were eligible were invited to complete the full survey. The survey consisted of tobacco use questions, loss aversion and delay discounting tasks completed via PsyToolKit, message exposure, questions regarding perceived message effectiveness, and demographic questions [31]. The full survey lasted approximately 17 min and participants were compensated with USD 1.75 via their MTurk account (USD 0.05 for the screener and USD 1.70 for the full survey). MTurk user IDs were tracked to ensure that individuals only participated once. At the end of the survey, participants were told that the messages were not approved by the FDA and were then provided with resources to help them quit smoking. This protocol (834419) was reviewed and considered exempted by the University of Pennsylvania IRB.

### 2.2. Data Collection

#### 2.2.1. Tobacco Use and Demographic Questions

Participants were asked to answer tobacco use and demographic questions. Tobacco use questions included those from the Fagerstrom Test for Nicotine Dependence (FTND) [32]. Participants were also asked “If you decided to give up smoking completing in the next 6 months, how sure are you that you would succeed” in order to gauge self-efficacy of smoking cessation (1 = “not at all sure” to 4 = “extremely sure”). Demographic questions asked participants for information regarding their gender, race, ethnicity, education, income, sexual orientation, employment status, marital status, and their state of residence.

#### 2.2.2. Behavioral Economic Tasks

Two behavioral economic tasks were included (1) a mixed gambles task to assess loss aversion and (2) a 5-choice discounting task to assess gain and loss delay discounting. Loss aversion and delay discounting tasks were completed in a randomized order prior to message exposure.

Participants indicated whether they would accept or reject gambles with equal odds of winning or losing hypothetical amounts of money in the mixed gambles task [33,34]. Delay discounting was assessed using the 5-trial adjusting delay discounting task [35]. In this discounting task, participants were presented with an amount of hypothetical money and asked if they preferred that amount in three weeks or half the amount now. Each of the subsequent four trials repeated this discrete choice question, but with titration up or down conditional on the previous response. Delay discounting was assessed for delayed gains and losses of USD 1000.

#### 2.2.3. Message Delivery

After completing the behavioral economic procedures, participants were randomly assigned to one of five message groups. The groups were based on a 2 (gain versus loss framing) × 2 (short-term versus long-term framing) between-subject design. A fifth group was included as a control condition in which participants were exposed to a message about television use; the use of an anti-smoking warning for the control message would not have been entirely separate from all other conditions given that warnings convey consequences (present or future) which must be conveyed as a potential loss or gain. In each experimental message group, participants were exposed to three messages (health, cost, and aesthetics, respectively) that represented one of the four experimental conditions or the control condition. Those messages are visible in Table 1. Long-term messages indicate more severe consequences than short-term messages because risks increase the longer an individual smokes cigarettes.

#### 2.2.4. Perceived Message Effectiveness

After exposure to the randomly assigned messages, participants were asked to respond to three statements related to perceived message effectiveness (PME) using the University of North Carolina’s PME scale [36]. Responses to each statement ranged from strongly disagree (1) to strongly agree (5). The three statements from UNC’s PME scale were: “This message discourages me from wanting to smoke”, “This message makes me concerned about the health effects of smoking,” and “This message makes smoking seem unpleasant to me”. Scores were summed for a total PME score variable.

### 2.3. Data Analysis

Descriptive statistics were first used to summarize study variables in the total sample and by randomized message type. Total PME scores were then evaluated first within the total sample using a one-way ANOVA with message type as the between-subject factor. Total PME scores were then evaluated within active message conditions using a 2 × 2 ANOVA with delay (short-term versus long-term framing) and sign (gain versus loss framing) as between-subject factors. Supplemental analyses also evaluated individual PME scores using this approach.

Individual difference predictors of PME response were evaluated using multiple regression within active message conditions. Demographic, tobacco use, and behavioral economic variables were included as predictors of PME total scores. Message type was also included in this model as two dummy-coded variables; (1) delay (long-term or short-term messages) and (2) sign (loss or gain related messages). Additional tests were conducted predicting individual PME items using this same approach. Similar results for these individual items were observed when analyzed using ordinal logistic regression models (Supplemental Table S1). Therefore, results are presented here for the linear regression models for conceptual simplicity and correspondence with total score analyses.

The impact of message exposure paired with behavioral economic variables was evaluated using median splits (defined as low versus high) on delayed rewarded discounting, delayed loss discounting, and loss aversion variables. Specifically, participants were considered congruent if these behavioral economics measures corresponded to those desirable for their randomized message condition (Table 2). A categorical rather than continuous split was used to relax a more rigid assumption of a linear impact of these behavioral economic matching variables on PME response. Sensitivity tests were also conducted considering congruency exclusively based on delay discounting or loss aversion variables rather than their combination. Analyses were conducted in *R* Statistical Analysis (R Foundation for Statistical Computing, Vienna, Austria) and JASP [37] using two-tailed tests and a type I error rate of 0.05. Effect sizes for pairwise comparisons are summarized using the effect size measure Cohen’s *d*.

## 3. Results

### 3.1. Sample Characteristics

Overall, participants (*n* = 512) were an average age of 40.7 (SD = 11.6), with a majority of the sample being female (62.2%) and White (88.9%). Participants reported smoking an average of 14.6 cigarettes/day (SD = 9.2) with an average FTND score of 4.6 (SD = 2.2). Table 3 contains demographic, tobacco use, and behavioral economic variables for the study sample separated by randomized message type. No significant group differences were observed for these variables, *p* values > 0.05.

### 3.2. Message Type

A one-way ANOVA indicated a statistically significant effect of message type, *F_*1,507*_* = 95.59, *p* < 0.001. This main effect primarily reflected significantly higher PME scores in the active message conditions compared to the control condition, *p* values < 0.001, Cohen’s *d* = 1.88–2.48 for active groups to control. The 2 × 2 ANOVA within active message conditions indicated a significant main effect of delay, *F_*1,407*_* = 11.26, *p* < 0.001. This effect was characterized by higher PME scores for messages containing long-term compared to short-term outcomes, Cohen’s *d* = 0.33. The main effect of sign, *F_*1,407*_* = 1.77, *p* = 0.19, and delay by sign interaction, *F_*1,407*_* = 0.22, *p* = 0.64, were not statistically significant. Similar results were observed for individual PME items (Table 4; significant effect of Delay *p* values < 0.02).

### 3.3. Individual Differences

Table 5 contains standardized regression coefficients (effect sizes) for individual difference predictors of PME total scores and individual items for the active message conditions. The previously described association of long-term (versus short-term) messages with higher PME scores was again observed within these multivariable models (i.e., when controlling for demographic, nicotine dependence, and behavioral economic variables). Furthermore, because gains and losses are valued differently in the present and the future, we examined both gain and loss discounting. Although delay discounting and loss aversion did not covary, gain delay discounting and loss aversion showed a relationship with perceived message effectiveness. Specifically, participants with shallower delayed reward discounting and lower loss aversion reported higher PME total scores, *p* values < 0.05. Older participants and those with greater perceived quit efficacy also had higher PME total scores, *p* < 0.05. These variables were each also associated with one or more individual item in the same direction with age significantly related to unpleasant and health consequence items, perceived efficacy significantly related to discourage and health consequence items, and low discount rates and loss aversion significantly related to the discourage item.

### 3.4. Congruency

**Figure 1:**
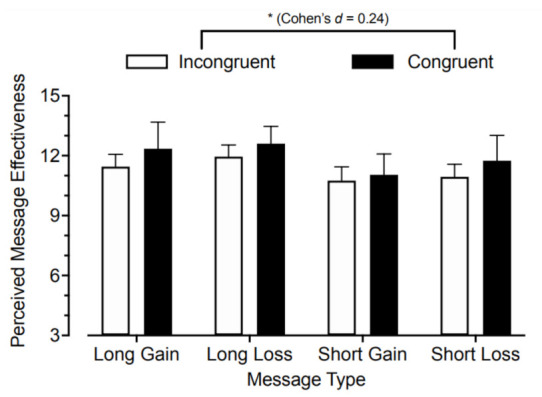
Total score perceived message effectiveness (PME) by message condition and behavioral economics matching. Presented are mean PME scores for each message condition. Error bars are 95% confidence intervals. Scores are divided by participants who were considered congruent versus incongruent with their randomly assigned message type based on behavioral economic delay discounting and loss aversion measures (see Data Analysis for details).

After random assignment to message exposure, approximately a quarter (23.3%) of participants happened to be congruent with a message condition best suited for their behavioral economic profile. A 2 × 4 ANOVA indicated a significant main effect of this congruency, *F_*1,399*_* = 4.14, *p* = 0.04, Cohen’s *d* = 0.24. This outcome reflected the higher total PME score for participants in a message condition congruent with their behavioral economic scores (Figure 1). The message type x congruency interaction was not statistically significant, *F_*1,399*_* = 1.39, *p* = 0.91, providing insufficient support for differential effects of the matching association between message conditions. These outcomes were selective to congruency determined by the combination of delay discounting and loss aversion, because a significant prediction was not observed when congruency was determined based on delay discounting, *p* = 0.40, or loss aversion, *p* = 0.10, alone. Analysis by individual PME item indicated the same pattern of effect for the discourage (main effect of congruency, *p* = 0.02, Cohen’s *d* = 0.27) and unpleasant (*p* = 0.03, Cohen’s *d* = 0.25) items, but no significant congruency effect for the health consequences item, *p* = 0.40.

## 4. Discussion

The purpose of the present study was to evaluate whether an individual’s loss aversion and delay discounting rates (i.e., behavioral economic characteristics) would influence the perceived effectiveness of anti-smoking messages through manipulation of the sign (gain versus loss) and delay (short-term vs. long-term) framing of those messages, particularly if message exposure was congruent with an individual’s behavioral economic characteristics. Smoking cessation messages were feasibly delivered through an online crowdsourced platform with greater perceived effectiveness compared to control messages. Greater perceived message effectiveness was observed for messages that described long-term compared to short-term outcomes, regardless of sign. Participants showing less devaluation of future rewards by delay and greater similarity in their sensitivity to positive and negative outcomes (i.e., lower loss aversion) reported higher overall perceived message effectiveness. Finally, participants who saw a randomized message that best addressed their behavioral economic response profile reported higher perceived message effectiveness, although of a smaller effect size than individuals whose randomized message exposure was not congruent with their behavioral economic preferences.

### 4.1. Effects of Messages

Participants who viewed experimental messages had higher PME scores than those exposed to the control condition. The evaluation of the main effects of these dimensions indicated no differential impact of gain or loss framing, but a statistically significant, small effect of long-term delay type. When examining group differences in sign alone, there was no difference between the gain and loss exposure groups. This finding may be attributable to the text-only nature of the messages. For example, Zhao and colleagues found that there was no framing effect when using text-only warnings, as this study did [19]. However, when examining the temporal context of the messages in the present study, there was a higher perceived effectiveness for those exposed to long-term messages compared to those exposed to short-term messages. This finding seems to counter temporal discounting research, which states that smokers tend to discount future outcomes more than non-smokers, and thus suggests a preference for short-term messages [24]. However, PME scores may be higher for long-term messages, because they indicate more severe consequences than short-term messages.

No interaction was observed by message type, indicating that the impact of sign did not differ by delay (or conversely that that impact of delay did not differ by sign). One reason for the lack of interaction may be attributable to the sign effect; this effect indicates that while the value of loss is discounted less over time, gains become increasingly less valuable in the future [38,39]. This effect can make it difficult to parse the effects of long-term messages. Another reason for the lack of interaction may come from the tense of the messages. In a study that examined the sign effect in past and future discounting, findings indicated that the tense in which the sign was presented has a multiplicative effect on the delay [40]. Specifically, the tense in which the sign of the message is conveyed can influence the amount of time one is willing to forego the consequence. In the current study, it is possible that the conditional tense of the messages—conveyed by words such as could and may—mitigated the value normally ascribed to potential health outcomes.

### 4.2. Effects of Individual Differences

Greater gain delay discounting and greater loss aversion were associated with lower PME scores. In terms of the gain delay discounting, this is an anticipated finding given that greater discounting of future gains has previously been associated with more severe nicotine dependence and reduced quit intentions/treatment responses [41,42]. However, higher rates of loss aversion related to reduced perceived message effectiveness is a novel finding. It may be that although the younger participants of the sample want to avoid potential losses, they have lower perceived personal health risks of smoking; this could explain why the messages are considered to be less compelling [43].

### 4.3. Congruency

Participants who were randomized to a message that was congruent with their behavioral economic profiles reported higher PME than individuals who were randomized to messages that were not congruent with their behavioral economic profiles, consistent with prospect theory. To date, no study has simultaneously tested how loss aversion and delay discounting rates of smokers can influence the perceived effectiveness of cigarette warning messages. We found that these behavioral economic differences influenced PME, which is a reliable measure of persuasiveness and has been associated with quit intentions and cessation behavior [44,45,46,47]. The current study offers evidence that tailored (congruent) messages improve PME, which could potentially lead to changes in smoking behavior. Through advances in technology, tailored messages are often used to target specific populations online. There is evidence that individualized public health communications have led to behavioral changes [48,49,50]. The tobacco industry and health agencies are already tailoring their respective messages using social media [51,52]. Further research should examine how these organizations might leverage behavioral economic profiles to increase the relevance of messages and the influence of individual behaviors. For example, a person who discounts the future at a high rate but has a low rate of loss aversion might find a message that states a more immediate, gain-focused consequence to be more effective than a message which conveys a long-term loss (as the currently proposed FDA warning messages are framed).

We are not aware of prior studies that have systematically evaluated loss aversion among smokers. The findings in this study indicate significant variation in the loss aversion rates of smokers. Instead of directing public health messages at the group level (i.e., smokers/nonsmokers), it may be advantageous to tailor messages according to individual-level loss aversion rates [53].

## 5. Conclusions

### 5.1. Limitations and Future Directions

At the time of writing, the ability of PME to predict behavioral change is debated; however, PME is a useful tool in determining whether subsequent behavioral studies are necessary [44,47,54,55,56]. Based on the findings, future studies should examine outcomes such as intention to quit or cessation. Additionally, messages in this study were only viewed once. Subsequent studies will rely on the Message Impact Framework to determine whether seeing congruent messages that are individually tailored multiple times might influence outcomes. Another limitation pertains to the inability to parse the magnitude of outcomes from the delay of outcomes, because the risk from smoking cigarettes is cumulative, becoming increasingly hazardous over time. Additionally, participants in the control group may be biased if they realized they were members of the control group based on messages unrelated to smoking.

### 5.2. Conclusions

Our findings highlight the influence of individual loss aversion and delay discounting. Rates on smokers’ perceived message effectiveness of cigarette warning messages, messages congruent with behavioral economic profiles, increased PME scores. The results suggest that tailored public health messages about smoking may be more beneficial than current anti-smoking warnings, which generally convey long-term losses. Additional research is necessary to determine the clinical significance of these findings.

## Figures and Tables

**Table 1:** Exposure group messages with health, cost, and aesthetic outcomes.

Exposure 1: Long-Term Gains	If you quit smoking cigarettes, you could add 10 years to your life. If you quit smoking cigarettes, your skin could look better for longer. If you quit smoking cigarettes, you could save thousands of dollars over 10 years.
Exposure 2: Long-Term Losses	If you continue smoking cigarettes, you could cut up to 10 years off of your life. If you continue smoking cigarettes, you could age prematurely. If you continue smoking cigarettes, you could spend thousands of dollars over 10 years.
Exposure 3: Short-Term Gains	If you quit smoking cigarettes, your breathing could improve in a month. If you quit smoking cigarettes, you wouldn’t smell like smoke. If you quit smoking cigarettes, you could immediately have more money for other things you enjoy.
Exposure 4: Short-Term Losses	If you continue smoking cigarettes, you could have trouble climbing stairs this month. If you continue smoking cigarettes, you could smell like smoke. If you continue smoking cigarettes, you are spending money that you could be using right now on other things you enjoy.
Exposure Control: Hours of Watching Television	Watching several hours of television could affect your health. There is a correlation between the number of hours that one watches television and one’s body fat mass. Time watching television could influence how you see the world.

**Table 2:** Congruent messaging given delay discounting and loss aversion rates.

Variable	Loss Aversion Coefficient		
Delay Discounting Rate		High Sensitivity to Loss	Low Sensitivity to Loss
	High Discounting Rate	Short-term, loss message	Short-term, gain message
	Low Discounting Rate	Long-term, loss message	Long-term, gain message

**Table 3:** Demographic, substance use, and behavioral economic variables by message assignment.

Variable	Control (*n* = 101)	Long-Term Gain (*n* = 102)	Long-Term Loss (*n* = 103)	Short-Term Gain (*n* = 102)	Short-Term Loss (*n* = 104)
	Mean (SD)/%	Mean (SD)/%	Mean (SD)/%	Mean (SD)/%	Mean (SD)/%
Demographic					
Age	39.9 (11.1)	41.6 (12.5)	41.5 (11.2)	40.0 (11.4)	40.7 (11.9)
Female	69.3%	57.8%	55.9%	61.4%	66.4%
Married	50.5%	52.0%	48.5%	46.1%	51.0%
White	90.1%	89.2%	89.3%	87.3%	88.5%
College Education	46.5%	45.1%	46.6%	52.0%	45.2%
Income ^a^	4.1 (1.8)	3.9 (1.8)	3.9 (1.7)	3.8 (1.8)	3.9 (1.9)
Substance Use					
Past Month Vaping	43.6%	43.1%	41.8%	42.2%	41.4%
Cigarettes/Day	14.2 (6.6)	14.9 (7.7)	15.2 (9.6)	14.7 (13.1)	14.1 (7.6)
FTND	4.4 (2.1)	4.7 (2.1)	4.7 (2.2)	4.5 (2.4)	4.8 (2.2)
Perceived Quit Efficacy ^b^	0.8 (0.9)	0.9 (1)	0.8 (0.8)	0.7 (0.8)	0.8 (0.9)
Behavioral Economic					
Gain Discounting (Log)	−2.4 (0.8)	−2.3 (0.8)	−2.4 (0.7)	−2.1 (0.9)	−2.3 (0.9)
Loss Discounting (Log)	−2.7 (1.2)	−2.7 (1.1)	−2.6 (1.3)	−2.9 (1.2)	−2.8 (1.2)
Loss Aversion	4.0 (3.0)	4.4 (3.3)	4.0 (3.2)	3.9 (3.4)	3.6 (3.2)

Note. FTND = Fagerström Test for Nicotine Dependence. ^a^ Income recorded as (0 = USD < 10k, 1 = USD 10k–15k, 2 = USD 15k–<25k, 3 = USD 25k–<35k, 4 = USD 35k–<50k, 5 = USD 50k–<75k, 6 = USD 75k–<100k, 7 = USD 100k–<150k, 8 = USD 150k–<200k, 9 = USD 200k+). ^b^ Perceived quit efficacy coded as (0 = Not at all sure; 1 = Moderately sure; 2 = Sure; 3 = Extremely sure).

**Table 4:** Main effect of delay on total PME scores and individual PME items.

Variable	Control	Long-Gain	Long-Loss	Short-Gain	Short-Loss
Total PME Score	5.5 (2.8)	11.6 (2.8)	12.1 (2.5)	10.9 (2.9)	11.1 (2.9)
Discourage from Smoking	1.8 (0.9)	3.6 (1.1)	3.7 (1.0)	3.5 (1.1)	3.4 (1.1)
Make Smoking Seem Unpleasant	1.8 (1.1)	3.9 (1.1)	4.2 (1.0)	3.6 (1.1)	3.9 (1.1)
Concerned about Health Consequences	2.0 (1.2)	4.1 (1.1)	4.2 (1.0)	3.8 (1.2)	3.8 (1.1)

**Table 5:** Individual predictors of perceived message effectiveness.

Variable	Total PME Score		Discourage		Unpleasant		Health Consequence	
	β	*p*	β	*p*	β	*p*	β	*p*
**Message Type**								
Long-Term Message	**0.14**	**0.004**	**0.10**	**0.046**	**0.12**	**0.015**	**0.15**	**0.003**
Loss-Framed Message	−0.05	0.259	0.01	0.826	**−0.12**	**0.015**	−0.03	0.513
**Individual Difference**								
Age	**0.13**	**0.016**	0.09	0.091	**0.12**	**0.029**	**0.12**	**0.021**
Female	−0.06	0.261	−0.05	0.348	−0.08	0.140	−0.02	0.629
Married	0.08	0.129	0.03	0.528	0.08	0.112	0.09	0.098
White	−0.06	0.214	−0.03	0.497	−0.03	0.561	−0.10	0.057
College Education	0.01	0.793	−0.01	0.853	0.04	0.379	0.00	0.976
Income	0.02	0.721	0.07	0.170	−0.06	0.264	0.04	0.490
Past Month Vaping	−0.06	0.245	−0.05	0.321	−0.04	0.413	−0.06	0.240
Cigarettes/Day	−0.04	0.496	−0.08	0.175	−0.01	0.908	−0.02	0.762
FTND	0.02	0.757	0.07	0.248	−0.01	0.835	−0.01	0.904
Perceived Quit Efficacy	**0.16**	**0.002**	**0.17**	**0.001**	0.09	0.067	**0.15**	**0.003**
Gain Discounting (Log)	**−0.11**	**0.030**	**−0.20**	**<0.001**	−0.09	0.103	−0.01	0.839
Loss Discounting (Log)	−0.06	0.258	−0.03	0.515	−0.07	0.198	−0.05	0.337
Loss Aversion	**−0.10**	**0.043**	**−0.13**	**0.015**	−0.06	0.225	−0.08	0.114

Note. PME = perceived message effectiveness. **Bold** = statistically significant associations.

## Data Availability

The data presented in this study are available on request from the corresponding author. The data are not publicly available in order to protect the privacy of participants.

## Supplementary Materials

The following are available online at https://www.mdpi.com/article/10.3390/ijerph181910492/s1, Table S1: Ordinal logistic regression results for individual items.
