# Supplementary material for: Tailored Cigarette Warning Messages: How Individualized Loss Aversion and Delay Discounting Rates Can Influence Perceived Message Effectiveness
Source: Int J Environ Res Public Health. 2021 Oct 6;18(19):10492. doi: 10.3390/ijerph181910492 (PMC8507605; doi:10.3390/ijerph181910492)
Supplement: Supplementary file 1 [file ijerph-18-10492-s001.zip › ijerph-1363800-supplementary.pdf]

International Journal of
Environmental Research and Public Health

MDPI

Supplemental Table S1. Ordinal Logistic Regression Results for Individual Items.

	Discourage		Unpleasant		Health Consequence	
	AOR	*p*	AOR	*p*	AOR	*p*
**Message Type**						
Long-Term Message	**1.47**	**.046**	**1.75**	**.004**	**1.97**	**.000**
Loss-Framed Message	1.12	.570	**0.62**	**.012**	0.94	.729
**Individual Difference**						
Age	1.02	.095	1.01	.100	**1.02**	**.047**
Female	0.88	.533	0.78	.223	0.98	.940
Married	1.20	.368	1.40	.089	**1.59**	**.021**
White	0.82	.531	0.85	.584	0.56	.064
College Education	1.01	.944	1.06	.752	0.92	.674
Income	1.06	.315	0.95	.312	1.06	.340
Past Month Vaping	0.88	.544	1.00	.983	0.95	.784
Cigarettes/Day	0.98	.112	1.00	.890	0.99	.488
FTND	1.08	.155	1.00	.989	1.01	.806
Perceived Quit Efficacy	**1.62**	**<.001**	1.24	.057	**1.41**	**.003**
Gain Discounting (Log)	**0.56**	**<.001**	**0.74**	**.020**	0.98	.906
Loss Discounting (Log)	0.93	.382	0.93	.384	0.93	.432
Loss Aversion	**0.92**	**.012**	0.96	.190	0.96	.150

Note. PME = perceived message effectiveness. **Bold** = statistically significant associations.
